# Application of Digital Image Correlation for Strain Mapping of Structural Elements and Materials

**DOI:** 10.3390/ma17112577

**Published:** 2024-05-27

**Authors:** Paweł Bogusz, Wiesław Krasoń, Kamil Pazur

**Affiliations:** 1 Institute of Mechanics and Computational Engineering, Faculty of Mechanical Engineering, Military University of Technology, gen. Sylwestra Kaliskiego 2 Street, 00-908 Warsaw, Poland; wieslaw.krason@wat.edu.pl; 2 Łukasiewicz Research Network—Institute of Aviation, al. Krakowska 110/114, 02-256 Warsaw, Poland; kamil.pazur@ilot.lukasiewicz.gov.pl

**Keywords:** strength of materials, experimental investigation, optical methods, DIC, digital image correlation method, strain gauge measurement

## Abstract

The strain gauge method and the digital image correlation (DIC) method are commonly employed for measuring strain in tested objects, including material specimens and structural elements. The optical method enables the assessment of 3D strain fields across the entire area of interest, achieved through cameras and advanced software. The study investigates both quasi-static strength tests and more advanced research of structures. It explores full-scale construction testing, featuring highly stressed components of new wagon designs. The paper reviews the benefits and challenges of using the DIC method to measure large-scale elements. Conducting full-scale object testing is characterized by significant complexity, often involving interactions between elements, complex loading conditions, and the influence of friction. Numerous factors affect the measurements. Therefore, to compare both methods, an initially standard shear by tensile test of CFRP composite was analyzed. The analysis of strain maps provides valuable visualization of deformation patterns occurring during construction loading. The strain gauge method was crucial for verifying the quality of the DIC measurements. The results obtained provide a detailed understanding of how the components behave, highlighting the versatility of digital image correlation technology. For strain values of 0.3% and above, a good match was obtained between optical and strain gauge measurements. Below this value, the results have less accuracy. The results obtained provide a detailed understanding of how the components behave, highlighting the versatility of digital image correlation technology. The error comparison and discussion between different measurement scenarios were conducted. The paper presents a developed methodology for measuring strain and displacement state in complex and crucial structural elements. The method can be applied to measurements of heavily loaded components used in the transportation industry; for example, in railways.

## 1. Introduction

The digital image correlation (DIC) method currently stands as the most significant non-contact strain measurement technique, and it is undergoing constant development [1,2,3,4]. It finds application in both laboratory and outdoor field testing. The 3D version of the DIC method holds particular importance, especially in the examination of metals, plastics, thermoplastics [5], advanced polymeric composites [6], and biological materials such as tissues [7], carotid arteries [8], balloons [9], and many others. This tool enables the conduct of quasi-static loading tests. Moreover, the increasing recording rates (up to tens of thousands of frames per second with reasonable resolution), CMOS sensors with short global shutter times, and high resolutions (up to 4 million pixels) of modern fast digital cameras make it a great choice even for dynamic measurements.

The DIC has replaced other non-contact optical techniques for conducting tests under complex loading conditions, such as the elasto-optic method, Moiré fringe projection method, speckle interferometry, holographic interferometry method, and grating interferometry. Nevertheless, sophisticated electronic versions of some of these methods, combined with computer analysis, continue to be employed.

For instance, electronic speckle pattern interferometry (ESPI) allows for determining the degree of deformation by analyzing the scattered laser beam reflected from a specially prepared rough surface of an object. The interferometry method is primarily applied in a range of strength tests, encompassing both static and fatigue conditions. It is commonly used in the research of specimens and components within the aviation and railway industries. The method enables the determination of stress concentration regions and the monitoring of the propagation of fatigue damage [10,11,12]. The laser grating interferometry method has been utilized in static and fatigue tests, including riveted and welded connections, as well as sandwich panel structures [12,13].

Based on a literature review, it is evident that the DIC method holds significant importance in the field of experimental mechanics [1,2,3,4]. The research scope includes the testing of new innovative railway elements [14]. It involves full-scale construction testing of highly stressed components of new railway designs. The tests were conducted on specially designed setups, mounted on a strength testing machine. Quasi-static compression and tension tests were performed on a prototype joint of a rotating platform railway wagon [14], with the joint components made of 41Cr4 steel.

Additionally, the study delves into the testing of advanced composite materials that can be utilized in these applications.

The paper is both research and review oriented, aiming to draw attention to the issues and challenges associated with measurements using the DIC method. In the following section, the DIC system is introduced, and its operating principles are briefly described. Subsequent chapters showcase selected experimental studies, both focusing on materials such as CFRP composites and full-scale steel structures. It serves as a continuation and summary of earlier research [15,16].

The primary objective was to measure changes in the strain field of the examined objects during loading, necessitating the application of modern strain measurement techniques. The DIC system was applied for this purpose, due to its high versatility. The implementation of new measurement techniques, regardless of their type, requires validation through widely recognized methods. To achieve this, comparative studies were conducted, involving the determination of strain component values in the same direction, and based on the same or similar measurement area using DIC (digital image correlation) and strain gauge techniques. Subsequently, digital image correlation measurements were compared with strain gauge measurements. The analysis of equivalent strain maps provided valuable insights into deformation patterns occurring during construction loading [10,17]. Issues related to the characteristics of both measurements were analyzed.

Conducting full-scale object testing is characterized by significant complexity, often involving interactions between elements, complex loading conditions and numerous factors can affect the measurements. Therefore, for a more precise comparison of both methods, the standard shear by tensile test of CFRP composite was analyzed.

Several publications, including [18], focus on studying the tensile properties of various materials using the digital image correlation (DIC) method. The literature and authors’ experience demonstrate that accurately measuring strain, even within the elastic range, is possible. The paper [19] presents a method for determining material constants using DIC, the finite element method, and the Gauss–Newton algorithm to optimize the results.

The novelty of the work is the application of a strain measurement methodology that combines the advantages of two different measurement systems: precise point measurements of up to 5% strain using strain gauges combined with the modern field measurement system of DIC. This methodology was used to evaluate the complex loading condition of a heavily loaded structure, specifically a railway wagon joint. No such comprehensive study on the simultaneous application of both measurement systems has been found in the literature.

## 2. DIC System Presentation

The basic principle of the DIC system is to track the same physical points represented as pixels in both the reference and deformed images. This involves selecting a square subset surrounding the tracked point in the reference image to locate the corresponding point in the deformed image. A predefined correlation criterion is established to evaluate the degree of similarity between the reference subset and its target subset. Due to the discrete nature of digital images, integer pixel displacements of the subset can be computed by implementing a searching scheme within the deformed image [20].

**Figure 1:**
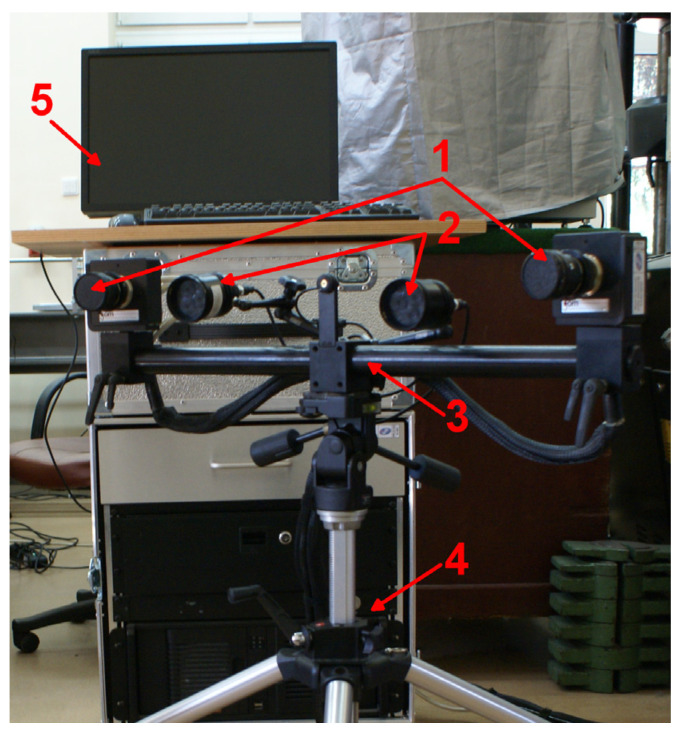
GOM Aramis system for DIC measurements: 1—two 4Mpx cameras for stereoscopic measurement; 2—LED lights illuminating the object; 3—camera support on the tripod; 4—computer PC with camera controller and dedicated software; 5—monitor.

The data presented in the paper were captured using the 4M DIC GOM Aramis system (Carl Zeiss GOM Metrology GmbH, Braunschweig, Germany) presented in Figure 1. This system is specifically designed for measuring deformations and strains on the surfaces of materials and structures under load. The key components of the DIC system are two high-resolution cameras (4Mpx) required for stereoscopic measurements in 3D, a set of calibration plates, and advanced GOM Aramis software v6.2.0-6 [21] for calculating strain tensors. The system allows for non-contact and full-field measurements.

A recorded image sequence undergoes processing using the digital correlation method. Displacement components are the fundamental data obtained. Based on them, equivalent Huber–von Mises–Hencky (von Mises strain) or directional strain components are obtained as needed. Users can define regions of interest for calculating the strain tensor [17,21].

The results are presented in the form of full-field maps and graphs, allowing for the recording and analysis of 3D deformation states of objects. Additionally, the system facilitates the export of results in the form of images and movies [21,22]. The DIC system can be used for both securing laboratory and outdoor testing in the quasi-static range [23], as well as for studying dynamically changing phenomena [24], which requires the use of high-speed cameras.

To ensure the accuracy of measurements, a calibration procedure utilizing calibration plates is essential. During calibration, the calibration plate is placed in several defined orientations, and the software recognizes and precisely measures the position of points marked on the panel. This recognition enables the system to determine crucial parameters of the DIC measurement, including the relative position of the cameras, lens distortion, and the intensity and uniformity of lighting. 

Accurate calibration is essential to achieve precise measurements. Through the calibration process, the system determines the measurement volume where precision is maintained, with a calibration deviation parameter ideally below 0.04 pixels [21]. Typically, the calibration deviation falls within the range of 0.02–0.03 pixels.

**Figure 2:**
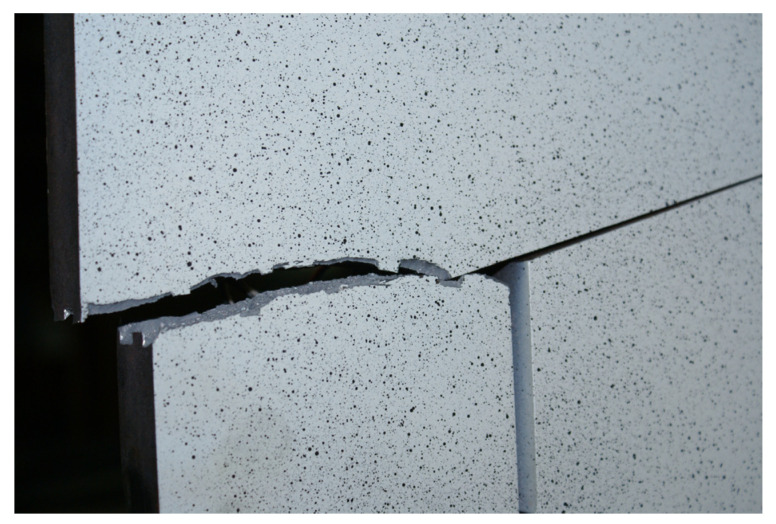
An example of a stochastic pattern applied to the elements of the tested railway joint.

Prior to conducting DIC measurement, a region of interest should be prepared in a specific manner by spraying a black and white speckle pattern, as shown in Figure 2. This configuration represents a typical pattern. Although it is possible to alter the pattern to a black background with white spots, experience suggests that the typical configuration is more effective.

The DIC procedure continues as follows. Following the preparation of the system and the research object in a testing bed, a test is initiated. At the start of the test, the DIC system captures a pair of images of the object in its initial state, usually before any external loading is applied. Upon loading, the observed surface moves and deforms. Subsequently, a sequence of image pairs is recorded throughout the loading process. The images depicting the object before loading function as a reference for computations during different loading states [10].

**Figure 3:**
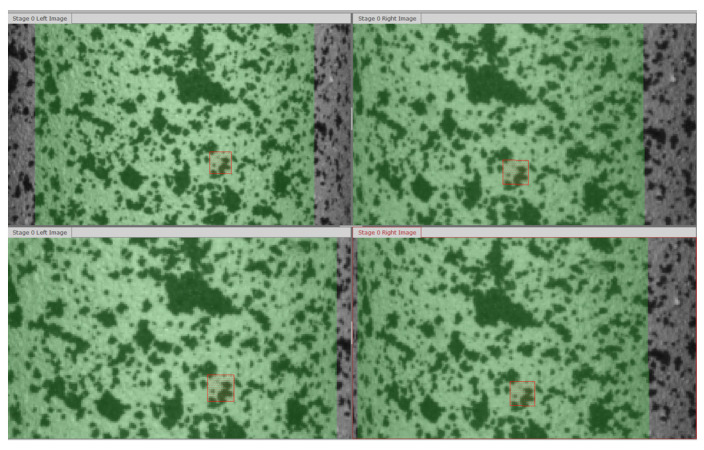
An example analysis of the virtual grid of facets in the DIC system using two subsequent pairs of pictures. The selected facet is highlighted by a red rectangle.

In the first image, a virtual regular grid of regions, referred to as facets, is applied to the stochastic pattern sprayed on the object (Figure 3). These facets, in the form of quadrilaterals, are then correlated with corresponding areas on images captured by the adjacent camera at the same stage, as well as on subsequent pairs of taken images. While the facet size is adjustable, the default size in the system is set to 15 × 15 pixels, with a default overlap of 2 pixels between neighboring facets.

Each region (facet) is characterized by a unique color scheme, represented by a grayscale histogram in the raw image. This color scheme is recognized in subsequent images of the recorded video. During the calculations, an analysis of the histograms of both the reference and current facet patterns is performed using the cross-correlation method in two perpendicular directions. The simplest image-matching procedure can be performed either in physical space using interpolations (nearest neighbor, bilinear interpolation) [2,23] or using a Fast Fourier Transform (FFT) to identify the best-matched regions [25,26]. A correlation compliance criterion is applied to assess the similarity between the reference and deformed images [10,21,27].

DIC system manufacturers often do not disclose detailed information about the software calculations and correlation algorithms used. However, examples of studies in the literature illustrate the specifics of these calculations. 

The concept of the DIC method is grounded in the principles of continuum mechanics [2,10]. The changes in dimensions and positions of short segments, determined by the positions of two points in the initial state and after deformation, are considered. These changes are described in a three-dimensional Cartesian coordinate system (3D). The deformation gradient tensor **F** is built. It connects the coordinates of deformed points $P_{\left(v,i\right)}$ with the coordinates of undeformed points $P_{\left(u,i\right)}$ (where *i* is an index for successive points). The connection of these coordinates is described by the relationship below [21]:

(1) $$P_{\left(v,i\right)}=u_{i}+F*P_{\left(u,i\right)},$$

where:

$P_{\left(u,i\right)}$—coordinates of undeformed points,
$P_{\left(v,i\right)}$—coordinates of deformed points,
$u_{i}$—translation of a rigid body.

The deformation gradient tensor can be decomposed into the rotation matrix **R** and the linear strain tensor **U**:

(2) F=R∗U,

where:

F—deformation gradient tensor,
R—right Cauchy–Green rotation tensor,
U—linear strain tensor.

To enhance the accuracy of DIC measurement, a specific sub-pixel registration algorithm is typically employed [20]. This involves approximating measurements of a few neighboring pixels through a continuous function, typically a polynomial function. 

Sub-pixel displacement determination can be achieved using the Gauss–Newton iterative least squares method, leading to a significant improvement in measurement precision. However, various other sub-pixel registration algorithms have been proposed in the literature. Among them, the iterative spatial domain cross-correlation algorithm using the Newton–Raphson or improved Newton–Raphson method is widely recognized as one of the optimal approaches. It is highly recommended for practical use due to its measurement accuracy and computational efficiency [20]. 

In 2004, Rogowska et al. [28] improved the speckle tracking algorithm by using a zero-mean normalized cross-correlation (ZNCC) criterion. An example of the zero-mean normalized cross-correlation criterion (ZNCC) is shown below [29]:

(3) $$C=\frac{\sum_{x=-N}^{N}\sum_{y=-N}^{N}\left[f\left(x,y\right)-\bar{f}\left(x,y\right)\right]\left[g\left(x\prime ,y\prime \right)-\bar{g}\left(x\prime ,y\prime \right)\right]}{\sqrt{{\sum_{x=-N}^{N}\sum_{y=-N}^{N}\left[f\left(x,y\right)-\bar{f}\left(x,y\right)\right]}^{2}}\sqrt{{\sum_{x=-N}^{N}\sum_{y=-N}^{N}\left[g\left(x\prime ,y\prime \right)-\bar{g}\left(x\prime ,y\prime \right)\right]}^{2}}},$$

(4) $$\bar{f}\left(x,y\right)=\frac{1}{{\left(2N+1\right)}^{2}}\sum_{x=-N}^{N}\sum_{y=-N}^{N}f\left(x,y\right),$$

(5) $$\bar{g}\left(x\prime ,y\prime \right)=\frac{1}{{\left(2N+1\right)}^{2}}\sum_{x=-N}^{N}\sum_{y=-N}^{N}g\left(x\prime ,y\prime \right),$$

where:

*f*(*x*,*y*)—grayscale value at the reference point (*x*,*y*),
*g*(*x′*,*y′*)—grayscale value at the current point (*x′*,*y′*),
$\bar{f}(x,y)$—average grayscale intensity value in the reference facet,
$\bar{g}(x,y)$—average grayscale intensity value in the current facet,
*N*–facet size.

The information about the relative position of the cameras computed during the calibration procedure enables the application of triangulation principles to determine the position of a point in the images from both cameras. These results are then compared with calculations obtained using the cross-correlation method. In the calculation phase, the overlay error is determined, aiming to be below the hundredths of a pixel.

The stochastic pattern applied to the surface of the measured object before the test, combined with photogrammetry and the cross-correlation method, allows for tracking shape changes and the displacement of each facet recognized in a region of interest on the loaded object. Maps of deformations and principal strains for the entire object are determined based on the displacement and deformation vectors of the region matrix [22]. However, it is also possible to obtain other strain-related quantities such as logarithmic strains, equivalent strains, shear strains, and strain rates.

According to the software documentation, deformation *λ* is defined as [21] follows:

(6) $$\lambda =\underset{l\to 0}{\lim}\frac{l+∆l}{l}.$$

The results presented in the paper show measurements of engineering strains, which are locally calculated using the following formula:

(7) $$\varepsilon =\underset{l\to 0}{\lim}\frac{∆l}{l}=\lambda -1.$$

The principal strains in optical measurements are determined based on the linear strain tensor U, related to the deformation gradient tensor F by the equation [21].

The matrix U is symmetric and can be transformed into a diagonal matrix. Its eigenvalues *λ*_1_ and *λ*_2_ are calculated based on mutually perpendicular directional strains, using the following expression [21]:

(8) $$\lambda_{1,2}=1+\frac{\varepsilon_{x}+\varepsilon_{y}}{2}\pm \sqrt{{\left(\frac{\varepsilon_{x}+\varepsilon_{y}}{2}\right)}^{2}-{\left(\varepsilon_{x}\varepsilon_{y}-\varepsilon_{xy}^{2}\right)}^{2}},$$

where:

ε_x/y_—directional DIC strains for the X and Y components, respectively.

The larger value allows for the calculation of the minimum strain, and the smaller value allows for the calculation of the maximum strain according to Formula (1).

The principal strains for the strain gauge method are determined based on strength formulas for rectangular strain rosettes, where strains are measured simultaneously and approximately pointwise in three directions, at angles of 0°, 45°, and 90°. The formula for major strain looks as follows [30]:

(9) $$\varepsilon_{max}=\frac{\varepsilon_{0{}^{\circ}}+\varepsilon_{90{}^{\circ}}}{2}+\frac{\sqrt{2}}{2}\sqrt{{\left(\varepsilon_{0{}^{\circ}}-\varepsilon_{45{}^{\circ}}\right)}^{2}+{\left(\varepsilon_{45{}^{\circ}}-\varepsilon_{90{}^{\circ}}\right)}^{2}},$$

where:

ε_0°/45°/90°_—rectangular rosette strains at angles of 0°, 45°, and 90°, respectively.

The formula for minor strain is presented below accordingly:

(10) $$\varepsilon_{min}=\frac{\varepsilon_{0{}^{\circ}}+\varepsilon_{90{}^{\circ}}}{2}-\frac{\sqrt{2}}{2}\sqrt{{\left(\varepsilon_{0{}^{\circ}}-\varepsilon_{45{}^{\circ}}\right)}^{2}+{\left(\varepsilon_{45{}^{\circ}}-\varepsilon_{90{}^{\circ}}\right)}^{2}}.$$

## 3. Tensile Test of CFRP Composite Material

To compare the DIC and strain gauge methods under predictable loading conditions, measurements were taken from a standard polymer composite. The study involved conducting shear-by-tension tests on six samples made of glass fiber-reinforced plastic (GFRP) composite.

The composite comprised glass fabric and vinyl ester resin. The BAT800 fabric was employed with an equal weight of fiber wefts and warps, equal to 400 g/cm^2^. VE-11M resin was used as the matrix material for the composite. The plate from which the samples were cut was produced using the vacuum infusion method, with a thickness of approximately 2.6 mm. The samples had a width of 25 mm.

The samples were manufactured and evaluated according to the guidelines outlined in standard PN-EN ISO 14129:2000 [31]. They were cut at a 45° angle to the weft and warp of the fabric, with the sequence of fabric layers relative to the plane of symmetry being [0/45/0/45]. The volumetric degree of reinforcement was 47.5%.

**Figure 4:**
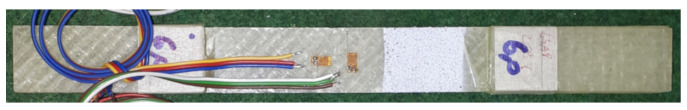
Prepared sample of the GFRP composite. Strain gauges in T-arrangement and DIC pattern visible.

Two linear strain gauges, each with a measurement area of 1.5 × 1.5 mm and a resistance of 120 Ω, were installed in the central measurement zone of each sample. The strain gauges were installed in a T arrangement (Figure 4), following standard requirements. For fabrics oriented at a 45° angle, this involved measuring shear strains while subjecting the sample itself to axial tension.

Some of the samples were subjected to testing with a stochastic pattern, as illustrated in Figure 4, which enabled the mapping of the strain field in the patterned area. In the case of DIC measurements, a stochastic pattern with dimensions of 25 × 35 mm was applied to the measurement area of the sample (shown in Figure 4). Strain measurements were then conducted to ascertain the shear curve and related strength properties. DIC strain measurements revealed the strain field of the tested material and were subsequently compared with strain gauge measurements.

Shear-by-tension testing of rectangular GFRP composite samples was carried out at an ambient temperature of 24 °C using an Instron 8802 universal strength testing machine (Illinois Tool Works Inc., Norwood, MA, USA). Shear strains were measured with strain gauges, and all data were synchronized with loading and DIC data using a ESAM Traveller CF strain bridge (ESA Messtechnik GmbH, München, Germany). The sampling frequency was set at 20 Hz. Due to optical measurements, tests were conducted at a constant displacement rate of 20 mm/min (the standard requires 2 mm/min). The rate was adjusted to align with DIC measurement capabilities.

It was assumed that we were dealing with orthotropic material. Furthermore, it was assumed that during the shear test, the composite material undergoes pure shear in accordance with the standard. Thermal effects were neglected.

**Figure 5:**
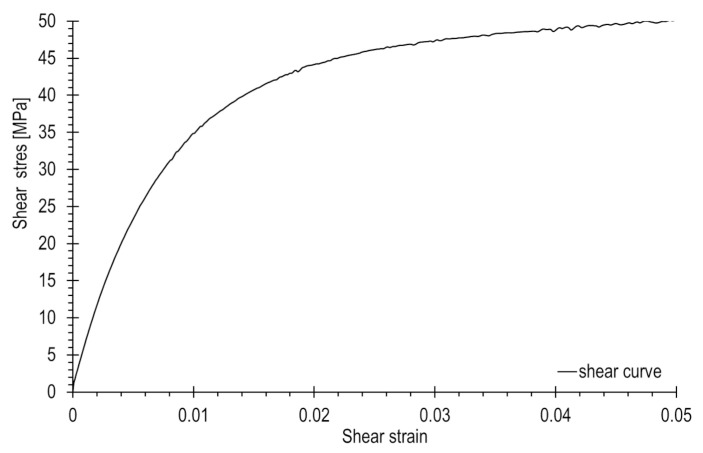
Shear stress-strain curve for GFRP composite, within the range of shear strains up to 5%.

Figure 5 illustrates a shear stress-strain curve, limited to shear strains of 5% according to the standard. Based on six conducted tests, an average shear curve and tensile strength parameters were determined: the tensile strength was equal to 49.5 MPa, and the shear modulus of elasticity was 4.0 GPa. The scope of GFRP composite studies was broad, encompassing tests at loading rates ranging from 0.02 to 200 mm/min, as well as creep tests. Further details can be found in [32].

**Figure 6:**
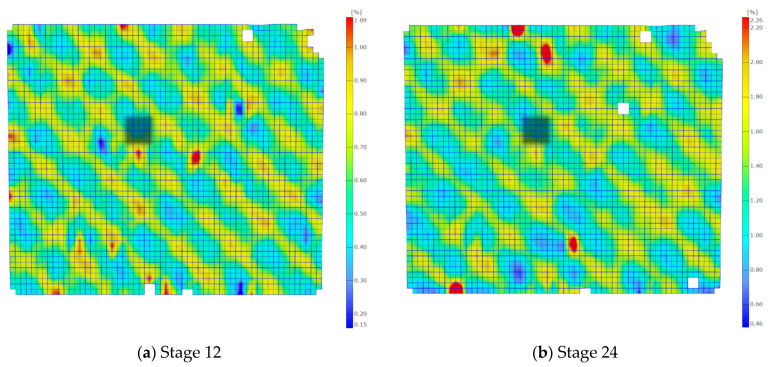

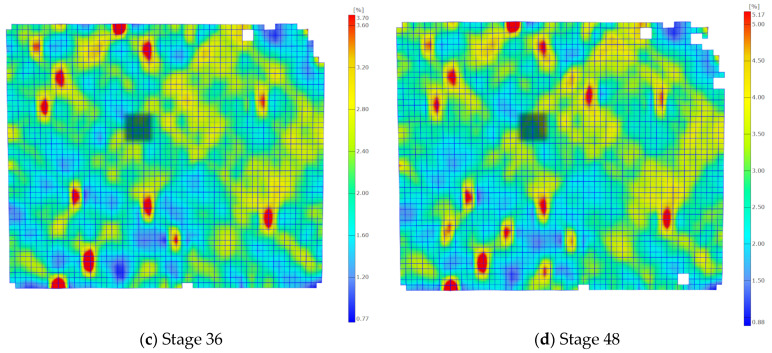
Maps of axial strains for selected stages of the test. The measurement area of the virtual strain gauge is marked with a gray rectangle.

In Figure 6, the evolution of the strain field during tensile loading is depicted through a sequence of four images taken every 12 frames, which cover a deformation range of up to 5%. The image capture frequency was set at 5 frames per second, resulting in images taken every 0.2 s.

The strain field map obtained from the stochastic pattern reveals non-uniformity, displaying traces of surface fibers in a net-like pattern on the sample. These traces become progressively blurred in subsequent sequences. The optical system enabled the measurement of the non-uniform distribution of the strain field caused by the layers of the glass composite fabric.

The maps of axial strains presented in Figure 6 correspond to the measurements of strain gauge installed parallel to the sample axis. To compare both measurement methods, the strains in the perpendicular direction were also obtained from the DIC results.

On the deformation maps, a gray rectangle is visible and marks the facets used for comparative measurements of the DIC and the strain gauge methods. In this range, the average values of axial and perpendicular strains, corresponding to the directions in which the linear strain gauges were installed, were defined. This includes the axial direction 0° defined as x and the perpendicular direction in the plane of the sample 90°, defined as y. Gamma strains were calculated using the same procedure as for the strain gauge measurements [31]:

(11) $$\gamma =\left|\varepsilon_{x}\right|+\left|\varepsilon_{y}\right|$$

where

ε_x/y_—strain measured by the DIC or strain gauge method in the axial or perpendicular direction, respectively.

**Figure 7:**
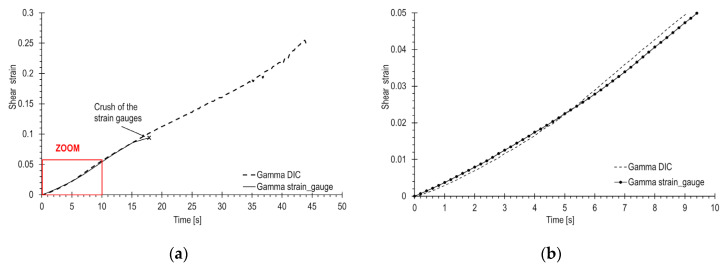
Comparison of shear strain in a GFRP composite sample determined by strain gauges and DIC: (**a**) across the entire range until failure, (**b**) zoomed within the 5% of gamma range.

Using Equation (11), the shear strain profile was determined through strain gauge measurements and the DIC method. The results are depicted in Figure 7. The first graph in Figure 7a) compares DIC and strain gauge strains across the entire range until failure, while Figure 7b) focuses on gamma strains zoomed within the 5% range. It was assumed that the strain field across the entire measurement section of the sample follows a consistent non-uniform distribution resembling a grid. Analyzing the strain maps in the DIC region of interest from Figure 6, it can be concluded that the assumption is valid and applicable to the entire measurement area of the sample.

The curves from Figure 7 exhibit similar shapes and characteristics. The average inconsistency between DIC and strain gauge measurements is 5.6%, with errors initially reaching several tens of percent at the beginning of the curve but quickly dropping to a range of ± several percent. It is important to note that these curves represent gamma values for various inhomogeneous measurement zones of the sample. The observation of a low scatter of DIC results is also noteworthy.

Although the standard [31] limits shear strains to 5%, the tests were carried out until the sample fractured. The corresponding gamma curves are depicted in Figure 7b). Optical measurements enabled the determination of gamma up to the point of crushing, whereas the strain gauges became damaged at a gamma strain level below 10%.

In this research, the optical method enabled precise measurement of the strain field in the CFRP composite until failure. Additionally, a non-uniform pattern in the measured strain field was identified.

## 4. Research of Full-Scale Steel Structures and Results

The following section covers aspects of testing full-scale objects under high loads and difficulties arising from the methodology and the nature of optical measurements in strength tests.

An experimental investigation into innovative solutions for railway components is presented [15]. The investigated constructions were evaluated with a specific emphasis on the strain and deformation of elements under high loads. Quasi-static compression and tension tests were performed on a prototype joint of the rotating platform railway wagon. Due to its versatility, the optical strain measurement method (Figure 2), as described in Section 2, was utilized in the conducted tests. Maps of directional strains, major/minor strains, and deformations for the joint components were determined. Strain values at selected points on the evaluated structures were verified using the strain gauge measurement.

### 4.1. Strain Measurement on Heat-Treated Steel Railway Joint

**Figure 8:**
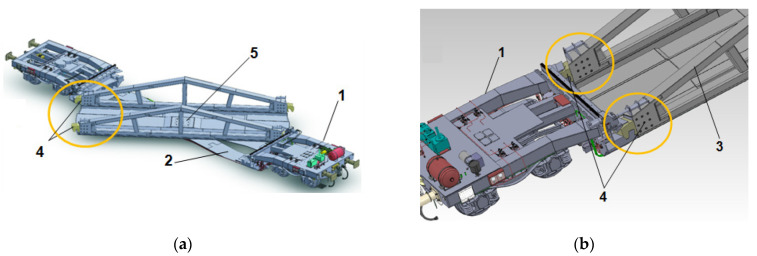
A prototype version of the wagon [14] with (**a**) open loading platform, (**b**) closed loading platform with the view of lock components: 1—over-bogie part of the wagon, 2—lowered fixed frame of the wagon, 3—side of the moving loading platform, 4—hook shape railway joint, 5—central node of the rotatable platform.

An intermodal railway wagon equipped with a low rotatable loading floor (Figure 8) designed for transporting truck vehicles by rail is the subject of the investigation. This structure is composed of a lightweight and lowered frame bottom (2), a rotatable floor in the body with a central node for transporting loads, and standard railway bogies (1). The wagon facilitates quick and efficient loading and unloading without the need for a platform infrastructure or terminals [14]. 

Based on the conducted FE analysis and strength tests [33] of the wagon, it was confirmed that the most critical components of the wagon with a rotatable loading platform are the side railway joints (4 in Figure 8a,b). These joints are part of a mechanism that locks the rotation of the loaded platform during transport. The design of such joints allows only the transmission of longitudinal load; thus, it does not block the rotation of the platform during loading/unloading operations [14].

The railway joint bears a heavy load during operation. Thus, the objective of the subsequent studies was to evaluate its behavior during load conditions. The railway joint is composed of two upper and lower elements made of heat-treated 41Cr4 steel, designed as irregularly shaped plates to ensure their cooperation. The upper joint plate had overall dimensions of 768 × 349 mm, while the lower element plate measured 815 × 400 mm. Both plates had a thickness of 30 mm. The joint elements were secured to the machine fixtures using 5–6 pins. 

**Figure 9:**
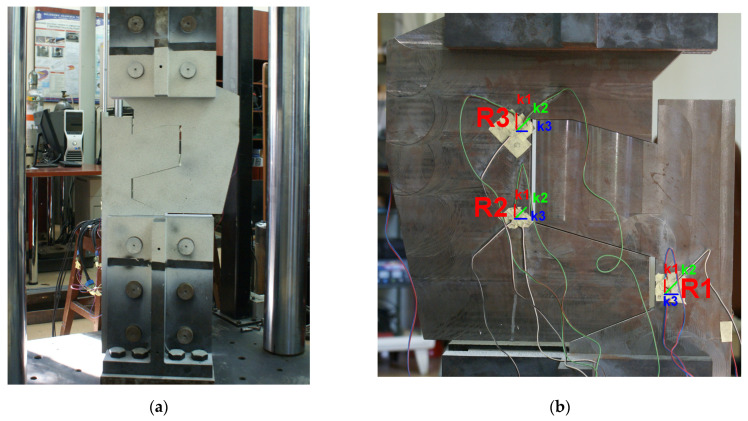
Railway joint on the test stand: (**a**) DIC region of interest with an applied stochastic pattern; (**b**) arrangement of strain gauges on the backside surfaces of the railway joint elements: R1-, R2-, and R3-rosettes. Directions labeling of the rectangular rosettes: k1—0°; k2—45°; k3—90°.

Static compression and tensile tests were conducted using the Instron SATEC testing machine (Illinois Tool Works Inc., Norwood, MA, USA) with a loading range of ±1200 kN. The loading rate was set at 10 mm/min, and the data sampling frequency was 10 Hz. The setup, along with the joint installed on the testing machine, is illustrated in Figure 9. The testing scenario involved two load-unloading cycles: compression (0-800-0 kN) and tension (0-500-0 kN).

Consistent with the earlier studies, optical DIC measurements were cross verified with strain gauges. Both measurement methods were conducted simultaneously and synchronized with each other, as well as with the applied load. Figure 9a presents the stochastic DIC pattern applied to the surface of the joint elements, while Figure 9b illustrates the arrangement of strain gauges installed on the opposite side of the joint.

In the electro-resistant method, three rectangular rosette strain gauges Vishay EA-60-060RZ-120/E (Vishay Precision Group, Inc, Raleigh, NC, USA). with a resistance of 120 Ω and gauge factor 2.07 were placed at locations expected to undergo high strains. They were installed on the surfaces opposite the DIC pattern on the railway joint (Figure 9a). The arrangement and orientations of the gauges are illustrated in Figure 9b. Rosettes were applied 12 mm from the cooperating lateral edges of both joint elements. On the lower element, a single rosette (labeled as R1) was attached, while on the upper element, two rosettes (R2 and R3) were installed. These rosettes were positioned so that one measurement direction was parallel to the load direction (k1), the second was set at a 45° angle (k2), and the third (k3) was perpendicular. 

**Figure 10:**
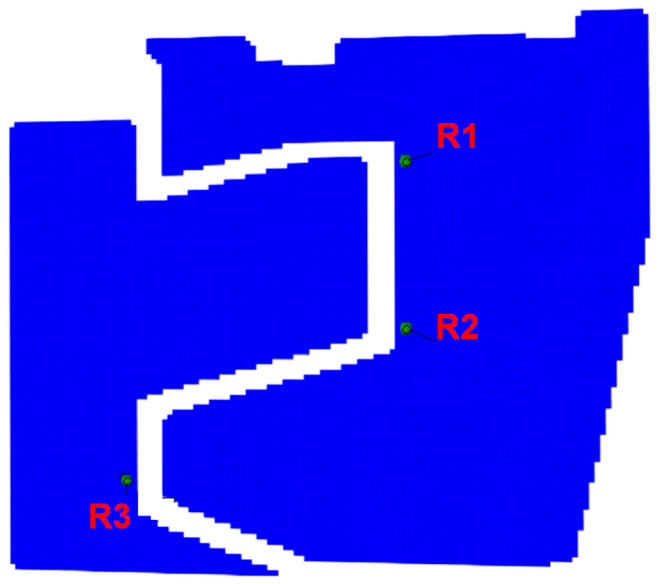
The arrangement of DIC measurement areas and their corresponding strain gauge sensors.

The optical system was configured for measurements using a 350 × 280 mm calibration plate, facilitating the calibration of a measurement volume of 500 × 370 × 500 mm, encompassing the tested railway joint elements. Lenses with a focal length of 50 mm were utilized. The cameras were positioned 1590 mm away from the front surface of the joint, resulting in a 678 mm distance between them. Images were captured at a frequency of 2 frames per second. The analysis involved facet fields measuring 20 × 20 pixels, with each facet having an area of approximately 3.5 × 3.5 mm. Figure 10 illustrates the selected facet fields and corresponding strain gauge sensor installation points.

### 4.2. Results of Strain Measurement on Railway Joint Elements

**Figure 11:**
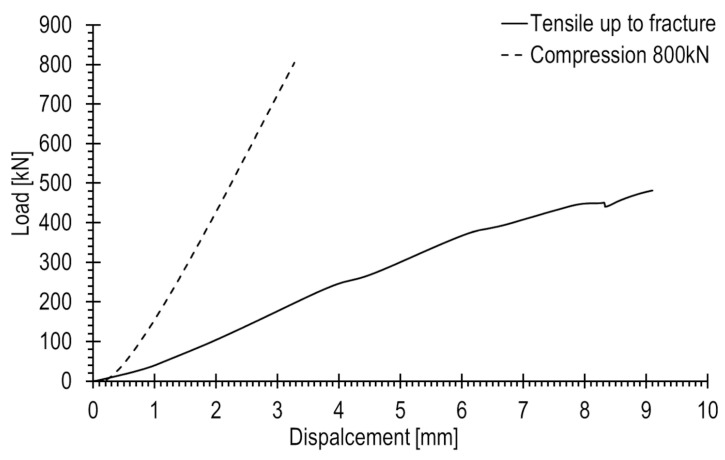
Load–displacement graphs determined at tension and compression of the railway joint.

The tension and compression curves of the joint elements, plotted in force-displacement coordinates, are compared on a shared graph in Figure 11. A significantly faster increase in compressive force is noticeable compared to tension. The maximum displacement at a force of 800 kN occurred at approximately 3.3 mm. During tension, the maximum force was achieved at a displacement of 9.1 mm. Significant deformations of the joint elements were observed, attributed to the asymmetrical design of the railway joint elements and primarily the conical arrangement of their interaction areas. Consequently, the bottom joint element sustained damage at a loading of 481.3 kN. The location of the fracture is depicted in Figure 2, highlighting its brittle nature.

**Figure 12:**
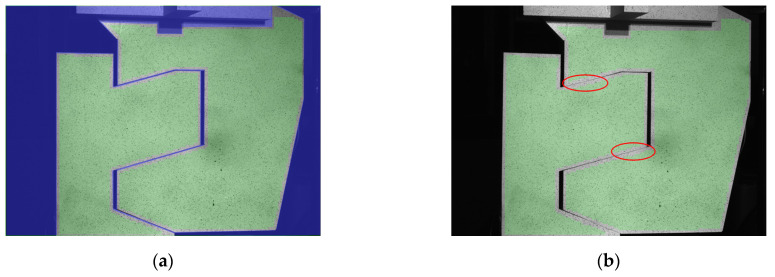
Pictures of the railway joint under compression: (**a**) the unloaded joint; (**b**) the joint loaded with 800 kN. The contact areas during compression were marked with red ellipses.

**Figure 13:**
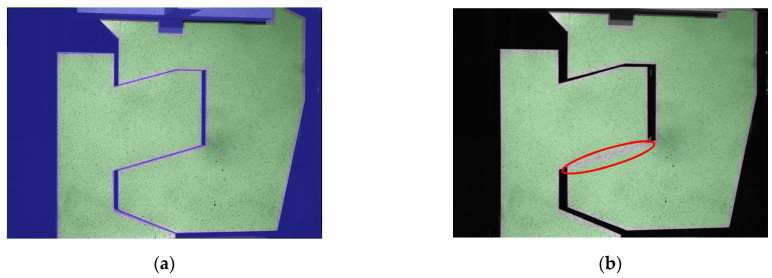
Pictures of the railway joint under tension: (**a**) the unloaded joint; (**b**) the joint loaded with 482 kN. The contact area during tension was marked with red ellipse.

Photographs of the railway joint elements subjected to compression and tension at the initial (a) and final (b) stages are shown in Figure 12 and Figure 13, respectively. The measurement area of the optical system is highlighted in green, while the regions where the joint elements interact with each other are clearly visible and encircled with red ellipses. In Figure 13b, a pronounced deflection of the upper element is depicted.

During the investigation, it was assumed that we were dealing with an isotropic and homogeneous material. The joint elements were subjected to tension and compression. However, due to geometric nonlinearities and the non-axial nature of the system, a state of complex loading occurs, resulting in corner stress concentration effects. Thermal effects were omitted due to the quasi-static nature of the loading and very low loading speeds.

**Figure 14:**
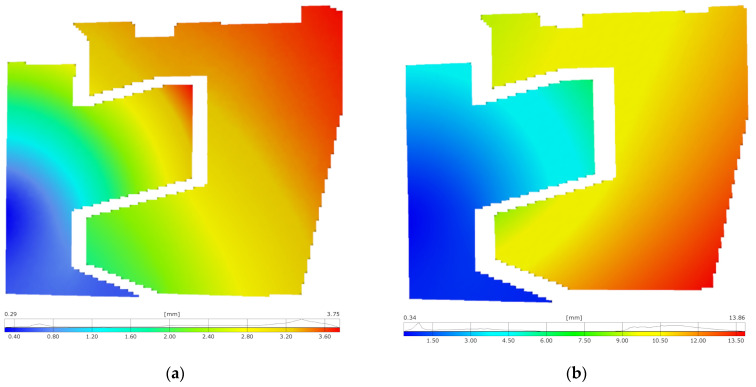
Maps of the total displacement of the joint elements under (**a**) compression and (**b**) tension.

Displacement maps (Figure 14), representing the total deformation, enabled qualitative and quantitative assessments of the joint elements’ behavior under axial loading, in both positive and negative directions. The deformation of the joint elements significantly differs between the two loading states.

In the compression test, the force increases more rapidly (Figure 11). The top outer corner of the upper element experienced the greatest deformation (Figure 14a). Lower displacement values were observed for the lower joint element, deforming only by 3.7 mm at its upper corner, where it contacts the upper element.

During the tensile test, the deformations of the joint elements are much greater. The bottom outer corner of the upper element moved the most, with its lower part shifting by 13.9 mm from the unloaded state (Figure 14b). The lower element did not deform as much, with significant deformations occurring at the point of interaction with the upper element, where a displacement of 5.8 mm was measured.

**Figure 15:**
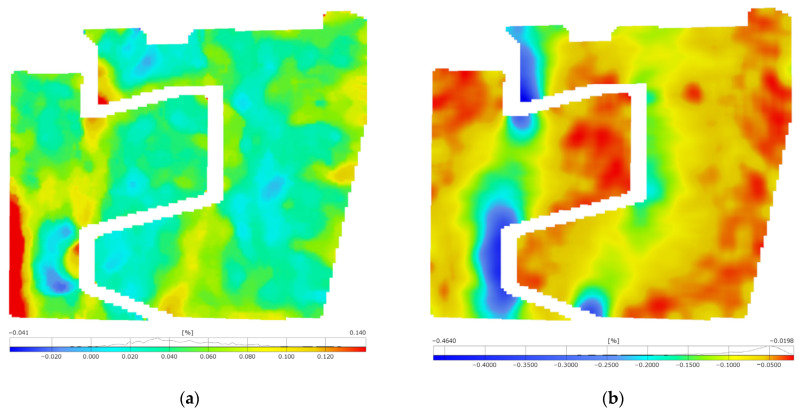
Maps of the principal strains in the joint elements subjected to compression within a range of 800 kN: (**a**) major strain;(**b**) minor strain.

**Figure 16:**
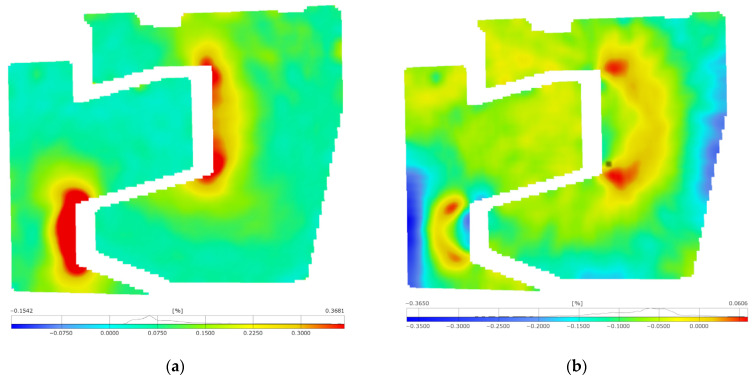
Maps of the principal strains in the joint elements subjected to tension up to fracture: (**a**) major strain; (**b**) minor strain.

In the following step, principal strain maps (Figure 15 and Figure 16) were determined, providing precise data on strain distribution and revealing strain concentration points in the railway joint elements, especially in their interaction areas. To achieve a clear distribution of strain maps and pronounce critical areas, it was necessary to filter the maps with a 3 × 3 facet averaging mask.

The strain maps from the compression and tensile tests reveal increased strains in areas where the elements of the railway joint come into contact. Significant major strains were observed during compression near the outer edges of the joint elements, especially the lower element and along the line of load application (Figure 15a). Major strains of 0.14% were recorded. The largest in magnitude minor strains (Figure 15b) revealed themselves along the axis of the joint loading, as expected. Their concentration occurs in the lower element, along the edge interacting with the upper element. Values exceeding 0.46% also indicate exceeding the yield strength. The presence of extreme values with opposite signs on the lower element indicates that it also underwent significant bending.

During the tensile test, significant strains were measured at the corners of cooperating surfaces, where tensile stress concentration occurred (Figure 16a). The outer edge of the lower element was subjected to compression, indicating that the entire lower joint element, except for tension, experienced bending. The maximum major strain exceeded 36% (Figure 16a), while the minimum minor strain was −36% (Figure 16b). Given that these are components for equivalent strain calculations, it can be concluded that the yield point was exceeded. The study highlights the importance of implementing rounded edges at the points where the components interact with each other.

However, it should be noted that the maps also present residual non-zero strains in areas where zero strain would be expected. For example, in the upper part of the lower element, strains of approximately 0.04% were recorded. This is within the limit of the method error.

**Figure 17:**
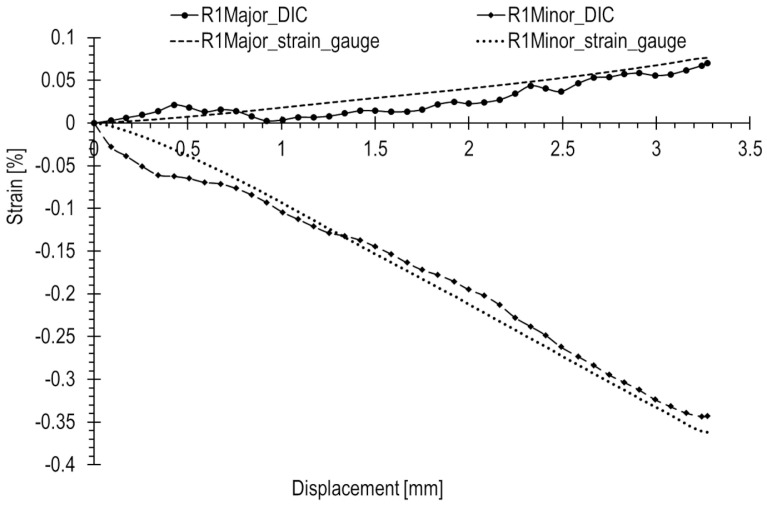
Principal strains measured using strain gauge rosette R1 and digital image correlation (DIC) during the compression of the railway joint.

**Figure 18:**
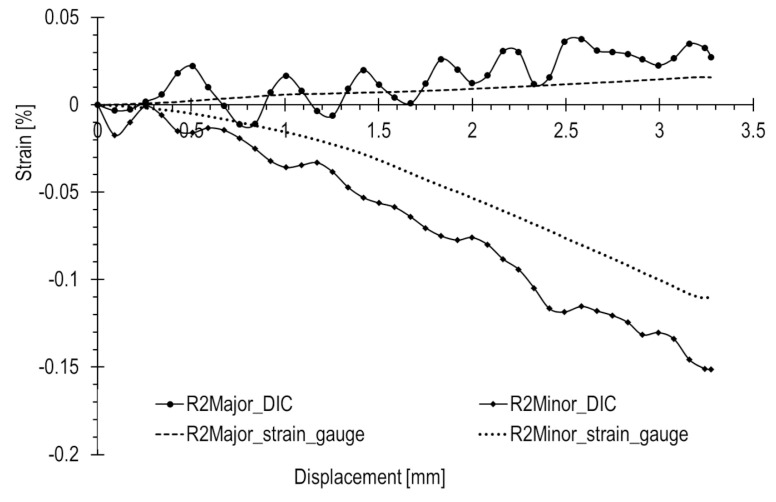
Principal strains measured using strain gauge rosette R2 and digital image correlation (DIC) during the compression of the railway joint.

**Figure 19:**
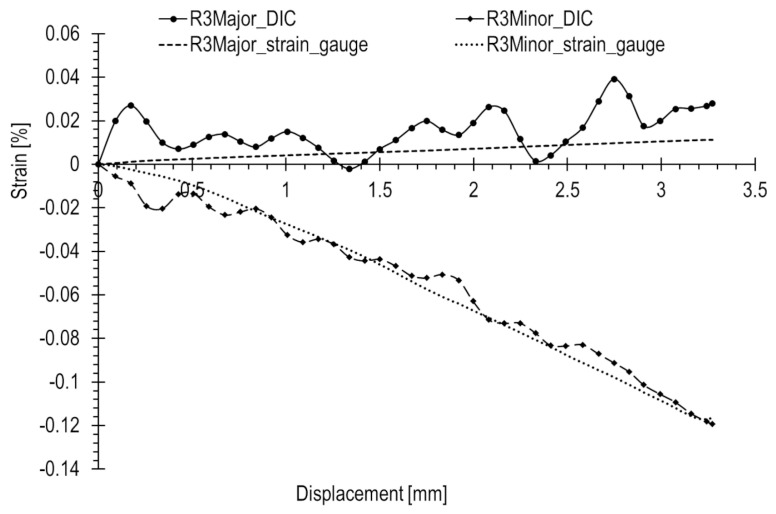
Principal strains measured using strain gauge rosette R3 and digital image correlation (DIC) during the compression of the railway joint.

The results of the R1–3 rosette measurements were compared to the results of optical measurements (Figure 17, Figure 18 and Figure 19, accordingly). To compute the corresponding data in the DIC system, facet fields were applied for both performed tests on the joint areas where the strain gauges were installed. However, it should be noted that facet fields were virtually placed in the DIC GOM Aramis software v6.2.0-6 (Figure 10) on the side of the joint with the stochastic pattern applied, while the strain gauges and rosettes were installed on the opposite side of the elements (Figure 9a,b).

The optical measurement graphs align with those obtained from the R1–3 strain gauges. Strain gauge readings exhibit an approximately linear trend, while the DIC readings fluctuate around linear approximations, showing a significant scatter of results, particularly in the initial segment. The most substantial differences are observed at the start of the curves. In general, there is a closer match between optical and strain gauge measurements for higher absolute strain values. However, for strains with lower values (below 0.05%), a notable spread in results is evident, as illustrated in Figure 18.

Table 1 presents a comparison of the principal strain values for all rosettes under a compression load of 800 kN, alongside corresponding values from strain gauges. The last column displays the relative error of the optical method in relation to the strain gauge measurement.

The R1 rosette recorded the highest absolute values of both major and minor strains under maximum compression, specifically 0.077% and −0.362%, respectively. This point is located near the inner wall of the lower joint element (Figure 10), in contact with the upper element during compression (Figure 13b). Similar strains were measured at the locations of the R2 and R3 rosettes, with a minor strain of 0.016% and a major strain of 0.11%.

In this loading scenario, major strains are notably low, below 0.05%, while the minor strains are significantly higher, reaching approximately 0.15% in absolute terms. The recorded error for major strains is consequently high, as the method error significantly influences the measured value. The maximal relative error reaches levels of 100% or higher. For measurements in the initial state, the error is even more pronounced, as illustrated by the graphs.

The possibility of such high measurement error values is due to the fact that the absolute measurement error of the method can reach approximately 0.2% [21]. In such cases, for low measured strain values, the percentage error is very high. This is particularly true for strains below 0.05%, such as minor strains.

Significant discrepancies in results and their spreads are attributed to the limited accuracy of optical measurements when dealing with exceedingly low strains. The method error is further amplified by the considerable distance of the cameras from the tested object (over 1.5 m) in relation to the considered dimensions (a few millimeters). Another practical challenge arises in correctly applying the stochastic pattern.

In the presented results, filtering with a 3 × 3 mask available in the DIC system and a moving average were successively applied. However, even with the use of more advanced signal filters, like wavelet filters, effectively eliminating measurement errors to ensure satisfactory alignment with the reference strain gauge signal remains challenging or even impossible. 

**Figure 20:**
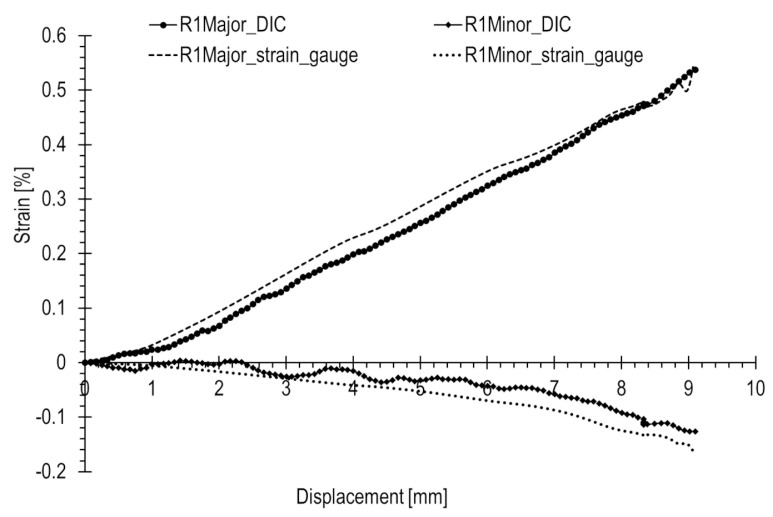
Principal strains from strain gauge (rosette R1) and DIC measurements during the tension of the railway joint.

**Figure 21:**
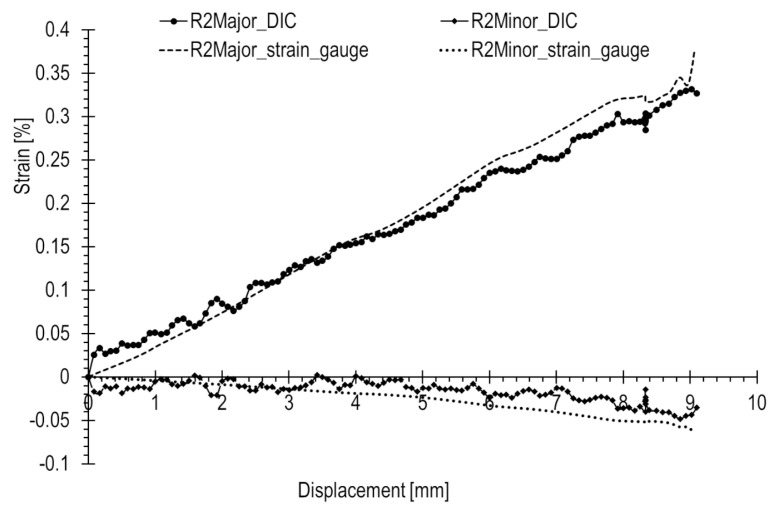
Principal strains from strain gauge (rosette R2) and DIC measurements during the tension of the railway joint.

**Figure 22:**
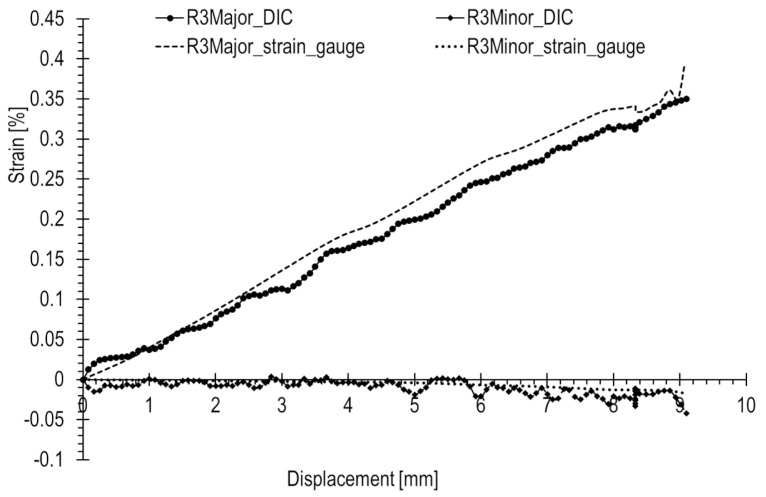
Principal strains from strain gauge (rosette R3) and DIC measurements during the tension of the railway joint.

Similar comparisons for the tensile test are depicted in Figure 20, Figure 21 and Figure 22. In this scenario, strains are generally higher than in the case of the compression state, where a rapid increase in loading force during compression occurs. Strains are consistently above 0.05%. Additionally, major strains are higher in magnitude than minor strains. Deformation is positive, and dimensions increase, leading to enhanced measurement precision.

Based on the results presented in Table 2 for the tensile test, the following was observed. The R1 rosette recorded at the point of failure the highest principal strains, with major and minor strains of 0.555% and −0.166%, respectively. Rosettes R2 and R3 also showed closely aligned strain values, measuring 0.4% and 0.02%, respectively. 

For strains with an absolute value of 0.3% and above, there a was good match (error not exceeding 15%) between optical and strain gauge measurements. However, below this threshold, the accuracy decreased with differences reaching several tens of percent. Notably, strains with an absolute value of 0.1% and below exhibited a considerable spread in results. 

At the ends of the major strain curves measured with the strain gauges rosettes, distinct non-linearities are observed. Considering the high precision of strain gauges, this may indicate the onset of yielding.

## 5. Summary and Conclusions

DIC and strain gauge measurements have their own advantages and disadvantages. Both methods require careful preparation, although the methodology and requirements for these preparations differ. 

The optical method is non-contact and non-destructive and allows for the measurement of strain, including large deformations. The DIC method creates strain maps on the structure’s surface within the designated area of interest. This is a significant advantage of the DIC system, as strain gauge measurement only allows for point measurements. It allows for the determination of stress concentration factors, yielding areas and the strain state in areas where installing a strain gauge is difficult or even impossible. Nevertheless, it has limited precision in the elastic strain range. The results obtained are influenced by many measurement parameters and the geometry of the measurement system (e.g., the angle and distance between cameras).

The strain gauge method is a point-based measurement, which allows for the measurement of strains usually up to about 5%. Its strong advantage is its precision in the elastic strain range. This method involves direct measurement and is therefore partially invasive. The strain gauge installation position must be prepared with the required precision.

Due to the challenge of determining measurement uncertainties for all factors affecting the measurement, and the limited precision of the measurement in the elastic range, the DIC method is often used in conjunction with another method. Most commonly, this involves strain gauge measurement.

Initially, the DIC was only used for static strength tests. However, the development of fast cameras has made it possible to use this method for dynamic testing. Recording speeds (up to tens of thousands of frames per second, with reasonable resolution), CMOS sensors with short global shutter times, and high resolutions (4 million pixels) of modern fast digital cameras allow for high-precision measurements of rapidly changing phenomena, such as crash tests. Attempts are being made to apply the DIC method to measure the strains of rotating elements. The authors currently focus on studying the deflections of helicopter blade models [34] and a marine propeller model [35].

The results of the study are outlined as follows:

- For strain values of 0.3% and above, a good match was obtained between optical and strain gauge measurements, with an error of no more than 15%. Below this value, the results have less accuracy. Differences can reach several tens of percent. A large variability was also noted for values of 0.1% and below.
- Due to the resolution of camera sensors, the DIC method is less precise than the strain gauge method and is dedicated to measuring larger strains, above which strain gauges are usually damaged (typically > 5%). The DIC gives fairly accurate results for strains greater than 0.3%.
- Generally, the character of optical measurement curves corresponds to the curves obtained by the more accurate strain gauge measurements. Significant differences and scatter in DIC results become noticeable in the curves of main strains below an absolute value of 0.1%. In such cases, filtering of data samples is necessary to improve the accuracy of the results.
- The optical system allowed for the measurement of the non-uniform distribution of strains (net-shape pattern) caused by the laminar structure of GFRP composite. The average difference in measurements between DIC and strain gauges is 5.6%. The error initially reaches several tens of percent at the beginning of the test but quickly decreases to the range of a few percent.
- Shear-by-tension tests of the GFRP composite were performed until the sample was fractured. The DIC measurements enabled the determination of the gamma curve until the sample’s crush, while the strain gauges were damaged at a shear strain level just below 10%.

## Figures and Tables

**Table 1:** Comparison of principal strains from a test at compression force equal to 800 kN.

Rosette	Principal Strain (Strain Gauge)		Principal Strain (DIC)		Relative Error	
	Major [%]	Minor [%]	Major [%]	Minor [%]	Major [%]	Minor [%]
R1	0.077	−0.362	0.070	−0.344	9.1	5.0
R2	0.016	−0.110	0.029	−0.151	−81.3	−37.3
R3	0.011	−0.117	0.026	−0.119	−136.4	−1.7

**Table 2:** Comparison of principal strains from a test at tension, before fracture.

Rosette	Principal Strain (Strain Gauge)		Principal Strain (DIC)		Relative Error	
	Major [%]	Minor [%]	Major [%]	Minor [%]	Major [%]	Minor [%]
R1	0.555	−0.166	0.538	−0.126	3.1	23.8
R2	0.382	−0.066	0.332	−0.048	13.2	27.0
R3	0.397	−0.018	0.350	−0.042	11.9	−132.7

## Data Availability

The original contributions presented in the study are included in the article, further inquiries can be directed to the corresponding author.
